# Multiplex real‐time PCR detection and relative quantification of periodontal pathogens

**DOI:** 10.1002/cre2.37

**Published:** 2016-08-11

**Authors:** Joshua Coffey, Mydah Choudhry, Marc Shlossman, Inder Raj S. Makin, Vineet K. Singh

**Affiliations:** ^1^ Missouri School of Dentistry and Oral Health Missouri USA; ^2^ Kirksville College of Osteopathic Medicine Missouri USA; ^3^ Arizona School of Dentistry and Oral Health, A.T. Still University of Health Sciences Arizona USA

**Keywords:** multiplex, periodontal pathogens, periodontitis, real‐time PCR

## Abstract

Periodontitis is a chronic inflammatory disease, which is strongly associated with certain pathogenic bacteria. The aim of this study was to develop a real‐time multiplex polymerase chain reaction (PCR) assay to detect and quantify bacterial species associated with periodontitis. We targeted detection and relative quantification of the following five bacterial species relevant to periodontal diseases: *Aggregatibacter actinomycetemcomitans*, *Fusobacterium nucleatum*, *Porphyromonas gingivalis*, *Treponema denticola*, and *Tannerella forsythia*. The conserved regions of the genome of these species were targeted with oligos and TaqMan probes in real‐time PCR assays. The species‐specific TaqMan oligos and TaqMan probes showed no cross‐amplification, and there was no loss of amplification yield in multiplex real‐time PCR assays. All five bacterial targets were amplified analogous to the template concentrations used in these assays. This multiplex real‐time PCR strategy could potentially be used to detect the bacterial species in periodontal pockets of patients with periodontal diseases. This assay may also serve as a quick tool for profiling and quantifying bacteria relevant to periodontal diseases and likely be a valuable tool for clinical translational research.

1

Periodontitis is a chronic inflammatory disease, of which specific bacterial pathogens are risk factors (Darveau, 2010; Socransky & Haffajee, 1992). During the development and progression of periodontitis, there is a well‐documented shift from a symbiotic microbiota to a dysbiotic community, from predominantly facultative Gram‐positive species to Gram‐negative anaerobes (Socransky & Haffajee, 1994). As these periodontal pathogens aggregate subgingivally, they inhibit the growth of other commensal species in the biofilm and work synergistically in pathogenesis (Thurnheer et al., 2014). These dysbiotic communities resist the host immune response and thrive in the inflammatory environment they stimulate (Moore & Moore, 1994). Increased inflammation causes collateral damage to the periodontium that eventually leads to tooth loss, making periodontitis the number one cause of tooth loss worldwide (Darveau, 2010).

Studies have implicated a variety of bacterial species in the development and progression of periodontitis (Perez‐Chaparro et al., 2014). Three of these species, *Porphyromonas gingivalis*, *Tannerella forsythia*, and *Treponema denticola*, are clustered in a group known as the “red complex” (Socransky et al., 1998). This red cluster antagonizes colonization of commensal bacteria and is considered the most pathogenic periodontal complex, found in significant numbers in active and advanced periodontitis (Thurnheer et al., 2014; Holt & Ebersole, 2005). *Fusobacterium nucleatum* is part of the “orange complex”, which is also associated with periodontal diseases (Socransky et al., 1998). *F. nucleatum* is a potent facilitator of periodontal aggregation (Jakubovics & Kolenbrander, 2010) and in some cases can constitute up to 20% or more of the bacteria in the subgingival biofilms (Suzuki et al., 2004). *Aggregatibacter actinomycetemcomitans* has been shown to be a potential etiological agent of an aggressive form of periodontitis particularly among adolescents from north and West Africa (Haubek et al., 2008). There are multiple serotypes of *A. actinomycetemcomitans* based on their cell surface polysaccharide antigens, and these serotypes demonstrate variable virulence, and some predominate in clinical samples (Brigido et al., 2014; Pahumunto et al., 2015). *A. actinomycetemcomitans* produces a cytolethal distending toxin that lyses neutrophils and plays a role in the breakdown of periodontal epithelium (DiRienzo, 2014). Overall, these five bacteria play important and significant roles in periodontal destruction.

It is thus obvious that the profile and prevalence of periodontal bacteria are different between patients with periodontal diseases compared with periodontally healthy subjects. Treatment of periodontal diseases to some extent is hampered by the lack of a rapid screening procedure for the prevalence of bacterial species in healthy and inflamed periodontal tissues. A rapid and sensitive multiplex real‐time polymerase chain reaction (PCR) assay has been optimized in this study to facilitate the detection and determination of relative abundance of bacterial species that are implicated in periodontal diseases.

## 2 MATERIALS AND METHODS

### 2.1 Bacterial strains and growth conditions

Bacterial strains and plasmids used in this study are shown in Table 1. *A. actinomycetemcomitans* (ATCC 43718), *F. nucleatum* subsp. *nucleatum* (ATCC 25586), *P. gingivalis* (ATCC 33277), *T. forsythia* (ATCC 43037), and *T. denticola* (ATCC 33521) were obtained from American Type Culture Collection (Manassas, VA, USA). Bacterial cultures were grown under anaerobic growth conditions at 37 °C using an anaerobic growth chamber AS‐580 (Anaerobe Systems, Morgan Hill, CA, USA) supplied with anaerobic gas (90% N_2_, 5% H_2_, and 5% CO_2_). *F. nucleatum* and *P. gingivalis* were cultured in brain heart infusion, *T. forsythia* was cultured using brain heart infusion supplemented with 2.5 mM N‐acetylmuramic acid (Sigma, St. Louis, MO, USA). *A. actinomycetemcomitans* and *T. denticola* were cultured in MTGE‐anaerobic enrichment broth (Anaerobe Systems).

**Table 1:** Bacterial strains and plasmids used in this study

Bacterial strains	Details	Reference
*Aggregatibacter actinomycetemcomitans*		ATCC number 43718
*Fusobacterium nucleatum*		ATCC number 25586
*Porphyromonas gingivalis*		ATCC number 33277
*Tannerella forsythia*		ATCC number 43037
*Treponema denticola*		ATCC number 33521
pGEM®‐T easy	*Escherichia coli* cloning vector	Promega
pGEM‐AA	pGEM®‐T easy with 637 bp DNA fragment specific to AA	This study
pGEM‐FN	pGEM®‐T easy with 653 bp DNA fragment specific to FN	This study
pGEM‐PG	pGEM®‐T easy with 637 bp DNA fragment specific to PG	This study
pGEM‐TD	pGEM®‐T easy with 600 bp DNA fragment specific to TD	This study
pGEM‐TF	pGEM®‐T easy with 600 bp DNA fragment specific to TF	This study

*Note*. AA = *Aggregatibacter actinomycetemcomitans*; FN = *Fusobacterium nucleatum*; PG = *Porphyromonas gingivalis*; TD = *Treponema denticola*; TF = *Tannerella forsythia*.

### 2.2 Genomic DNA isolation, PCR amplification of specific DNA targets and cloning

Genomic DNA was isolated from cultured bacterial cells using GenElute^TM^ bacterial genomic DNA isolation kit (Sigma) as per manufacturer's recommendations. The following genomic regions were targeted for designing oligos: *A. actinomycetemcomitans* (23S ribosomal RNA gene), *F. nucleatum* (16S ribosomal RNA gene), *P. gingivalis* (23S ribosomal RNA gene), *T. denticola* (16S ribosomal RNA gene), and *T. forsythia* (chaperonin *groL* gene). Oligos were designed using Beacon Designer^TM^ 8 (Premier Biosoft) program, and these oligonucleotides were obtained from Eurofins (Huntsville, AL, USA). Specific forward and reverse oligos (shown in Table 2) were used to amplify 600–700 bp fragments from the target regions mentioned previously for each of the bacterial species, and the amplified DNA fragments were cloned in the cloning vector pGEMT^Easy^ (Promega, Madison, WI, USA) (Table 1). The authenticity of these cloned DNA fragments was verified by PCR using TaqMan oligo pairs internal to the cloned fragments (data not shown).

**Table 2:** TaqMan oligos and TaqMan probes used in the real‐time assays

Targets	Sequence (5′→3′)	Description	Amplicon size
AA	TACTAATTAAGTGGGAAA	Forward cloning oligo	637 bp
	ATCTCTCAGTGTTAATAG	Reverse cloning oligo	
	GCGAAACGAAGAGAAGCAAG	TaqMan forward oligo	111 bp
	CCTACCCAACAGGCGTATCA	TaqMan reverse oligo	
	ATTCCCAACCGCACTT	TaqMan probe with 3′ BHQ 1 and 5′ 6‐FAM	
FN	CAACACCTAGTAATCATC	Forward cloning oligo	653 bp
	CGAATGCTAATACCTATA	Reverse cloning oligo	
	GGCTTCCCCATCGGCATTCC	TaqMan forward oligo	123 bp
	AATGCAGGGCTCAACTCTGT	TaqMan reverse oligo	
	TCCGCTTACCTCTCCAG	TaqMan probe with 3′ BHQ 2 and 5′ Cy5	
PG	GGAGAACCTACTGGAAAG	Forward cloning oligo	637 bp
	GGAGTTTATCTGGACTTGA	Reverse cloning oligo	
	CTGCGTATCCGACATATC	TaqMan forward oligo	134 bp
	GGTACTGGTTCACTATCG	TaqMan reverse oligo	
	ACCATAGACGACGGAGCACC	TaqMan probe with 3′ BHQ 2 and 5′ Texas Red	
TD	AAAGGTTGTAAAATTCTT	Forward cloning oligo	600 bp
	CCATATCTCTATGTCATT	Reverse cloning oligo	
	GTTGTTCGGAATTATTGG	TaqMan forward oligo	109 bp
	GATTCAAGTCAAGCAGTA	TaqMan reverse oligo	
	TCACACCAGGCTTACC	TaqMan probe with 3′ BHQ 2 and 5′ Cy5.5	
TF	GTTAAGGTAACATTAGGT	Forward cloning oligo	600 bp
	TTTATCGTAGATCAGAAT	Reverse cloning oligo	
	GAGGTTGTGGAAGGTATG	TaqMan forward oligo	108 bp
	GTAGATCAGAATGTACGGATT	TaqMan reverse oligo	
	TCTCCGCTTATTTCGTGAC	TaqMan probe with 3′ BHQ 2 and 5′ HEX	

*Note*. Oligos based on published sequences.

AA = *Aggregatibacter actinomycetemcomitans;* FN = *Fusobacterium nucleatum*; PG = *Porphyromonas gingivalis*;TD = *Treponema denticola*; TF = *Tannerella forsythia*.

### 2.3 Optimization of oligos and real‐time PCR conditions

Specific forward and reverse oligos internal to the DNA targets cloned in the vector pGEMT were used in real‐time PCR optimization assays. A 10‐fold dilution series was prepared for each plasmid DNA starting at 50 pg and used as template in real‐time PCR assays to determine amplification efficiency and reproducibility. The singleplex SYBR Green‐based real‐time PCR was performed using IQ5 real‐time thermocycler (Bio‐Rad). The reaction mixture (total 25 μL) contained 12.5 μL of 2× SYBR Green PCR master mix (Bio‐Rad) and 150 nmol of forward and reverse oligos and 1.0 μL of plasmid DNA dilutions described previously as template. The PCR amplification conditions were as follows: 40 cycles of 95°C for 15 s and 55°C for 30 s with an initial cycle of 95°C for 3 min. All amplifications and detections were carried out in a Bio‐Rad optical 96‐well reaction plate with optical sealing tapes (Bio‐Rad). Data were analyzed using the Bio‐Rad software integrated with the thermocycler.

### 2.4 Optimization of oligo specificities

Once the oligo efficiency was established and PCR conditions were optimized with plasmid borne DNA as template, the forward and reverse TaqMan oligos were mixed in equimolar concentrations. The mixture of oligos was then used in distinct real‐time PCR assays with genomic DNA isolated from each of the five bacterial species of interest as template. The reaction mixture included 12.5 μL of 2× SYBR Green Master Mix, the oligo mix (150 nmol of each of the five forward and reverse TaqMan oligos), and individual bacterial genomic DNA (1 ng) as template. In parallel assays, each forward and reverse bacterium‐specific TaqMan oligo pair was used in an amplification using a mixture of genomic DNA (5 ng; a mixture containing 1 ng of each of the five bacterial genomic DNA). These two amplifications were compared with a control amplification, which consisted of identical concentrations (1.0 ng) of individual bacterial genomic DNA and individual bacterium‐specific forward and reverse TaqMan oligos.

### 2.5 Optimization of TaqMan probes and multiplex real‐time PCR conditions

The TaqMan probes used in this study with the reporter dye and quencher are shown in Table 2 and were obtained from Eurofins. The TaqMan probes were initially optimized in a singleplex assay using Bio‐Rad CFX96 touch thermocycler. These assays were carried out in a total reaction volume of 50 μL that contained 25.0 μL of the 2× universal probe mix (Bio‐Rad), 150 nM each of the forward and reverse TaqMan oligos (Table 2), 150 nM of the TaqMan probe, and 50 fg of the target DNA on the plasmid pGEMT (Table 1). The PCR amplification conditions were as follows: 40 cycles of 95°C for 15 s and 55°C for 30 s with an initial cycle of 95°C for 3 min. Data were analyzed using the Bio‐Rad software integrated with the thermocycler. A multiplex PCR assay was also carried out that included all five forward and reverse TaqMan oligos and probes at 150 nM concentrations and a mixture containing 50 fg of each target DNA as template. PCR amplification conditions were the same as outlined previously. In these assays, the *C*_*q*_ and RFU values (representing the extent of amplification) were compared for specific targets between singleplex and multiplex assays.

## 3 RESULTS

### 3.1 Optimization of oligos and real‐time PCR conditions

The efficiency of the oligos intended to be used in a multiplex reaction was initially assessed in an SYBR Green‐based amplification using plasmid borne DNA as template. Decreasing concentrations of forward and reverse oligos (600–75 nM concentration) were used in singleplex PCR assays. In these assays, an oligo concentration of 150 nM was determined to provide the highest RFU values representing the amount of amplified DNA. An increase in the amount of the oligo concentration did not increase the amount of amplified DNA with 0.5 pg plasmid DNA as template, but a reduced amount of oligo concentration reduced the amount of amplified DNA (data not shown). We also investigated various annealing temperatures to determine one optimum annealing condition for best amplification for all five bacterial targets (data not shown).

**Figure 1:**
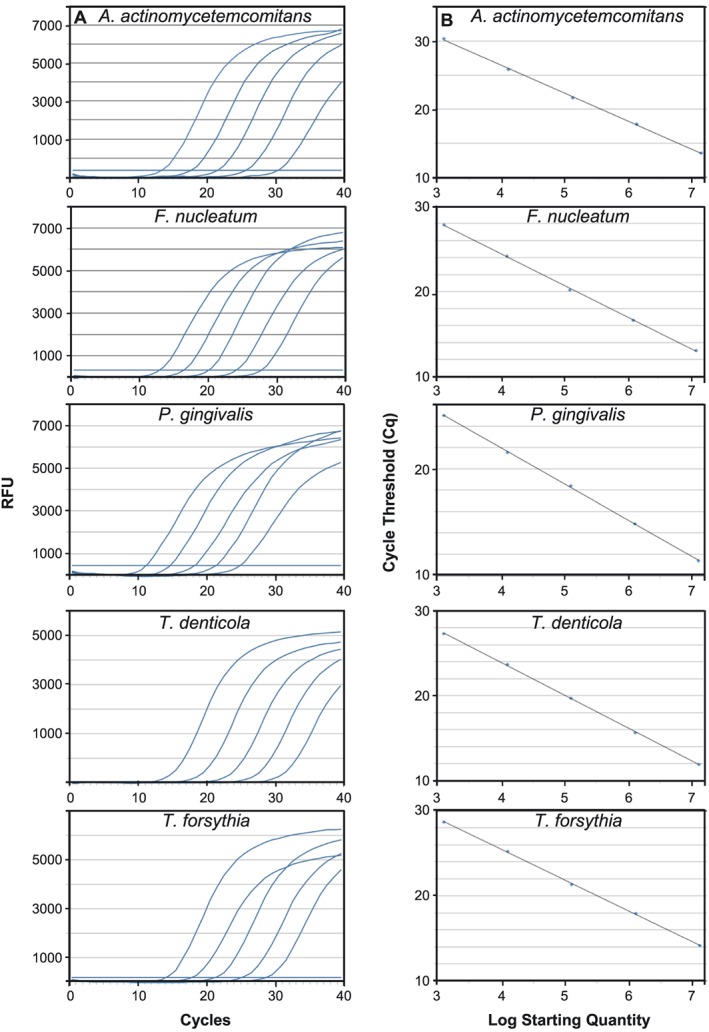
SYBR Green fluorescence during real‐time amplification with species specific oligos: (a) A gradual delay in amplification and an increase in *C*_*q*_ are apparent, which is consistent with 10‐fold gradual decrease in template concentration. (b) The standard curve representing the dilution series indicated in “A”. The *C*_*q*_ signal is titrated from 0.5 fg to 50 pg and shown in the standard curve. The slope indicates high level of polymerase chain reaction efficiency

Subsequently, the amplification efficiency was investigated using the optimized oligo concentrations and with decreasing concentration of plasmid borne template DNA. In these assays, the oligos amplified target DNA in a template concentration‐dependent manner for all five bacterial species (Figure 1). The amplification efficiency ranged from 73.7% to 97.0% with *R* ^2^ values close to 1.0 for all amplifications (Table 3). PCR assays should typically have high‐efficiency values and an *R* ^2^ value of 0.98 or above (Nolan et al., 2006). Although the *E* values for *A. actinomycetemcomitans* and *T. denticola* (Table 3) are a bit lower, the *C*_*q*_ values showed a consistent decrease when the template concentrations were raised in the PCR assays. As can be seen in Table 3, the *R* ^2^ values for all five oligo sets represent highly consistent and reproducible assays. We decided to use these oligo concentrations and PCR conditions in all subsequent assays.

**Table 3:** Efficiency of TaqMan oligos with bacteria‐specific DNA template

Oligos	*E* value	*R* ^2^ value
*Aggregatibacter actinomycetemcomitans*	73.7%	0.999
*Fusobacterium nucleatum*	85.9%	0.999
*Porphyromonas gingivalis*	97.0%	1.000
*Treponema denticola*	80.9%	1.000
*Tannerella forsythia*	88.0%	1.000

*Note*. *E* value indicates polymerase chain reaction (PCR) efficiency; an *R* ^2^ ≥ 0.98 indicates a reliable and reproducible PCR assay.

### 3.2 Optimization of oligo specificities

**Figure 2:**
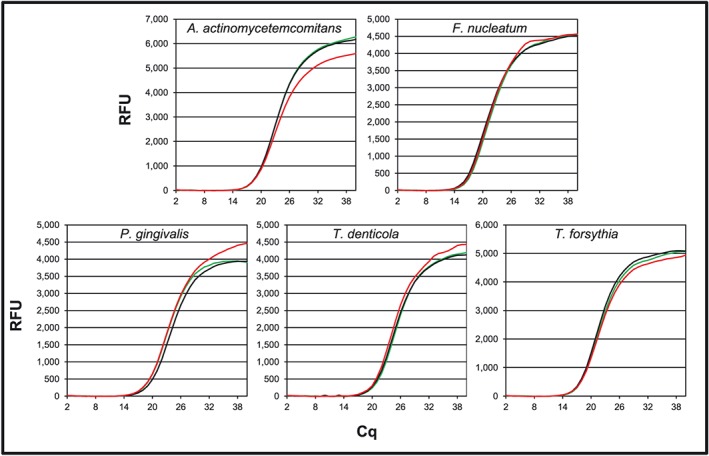
Real‐time amplification demonstrating oligo specificities: SYBR Green fluorescence during real‐time amplification with species‐specific TaqMan oligos and genomic DNA (black lines), bacteria‐specific TaqMan oligos with a mixture of bacterial genomic DNA as template (green lines), and mixed TaqMan oligos and individual genomic DNA as template (red lines). The amount of each oligo and each genomic DNA concentrations was identical whether included individually or in mixture

A three‐way amplification was carried out to measure oligo specificities. In these assays, the amplification observed with mixed oligos and pure genomic DNA or species‐specific oligos with mixed DNA was comparable with the amplification with bacteria‐specific genomic DNA and oligos (Figure 2; Table 4). The mixture of oligos amplified a single DNA target when genomic DNA from one bacterial species was used as template based on melt curve analysis (data not shown). A single DNA target was also amplified when the mixed genomic DNA was used as template with oligo pairs specific for a single bacterial species (data not shown). The comparison of amplification in conjunction with melt curve suggested lack of interactions between oligos and non‐specific DNA targets. These PCR assays suggested specificity of the oligos for their respective bacterial species and PCR conditions.

**Table 4:** Specificities of TaqMan oligos using pure and mixed bacterial genomic DNA as template

Conditions	AA	FN	PG	TD	TF
Individual template + individual oligos	19.60	19.57	19.78	21.53	17.93
Mixed template + individual oligos	18.62	17.18	20.24	21.26	17.73
Mixed oligos + individual template	18.79	17.50	19.58	20.94	17.92

*Note*. Numbers indicate *C*_*q*_ values.

AA = *Aggregatibacter actinomycetemcomitans*; FN = *Fusobacterium nucleatum*; PG = *Porphyromonas gingivalis*; TD, *Treponema denticola*; TF = *Tannerella forsythia.*

### 3.3 Optimization of TaqMan probes and multiplex real‐time PCR conditions

**Figure 3:**
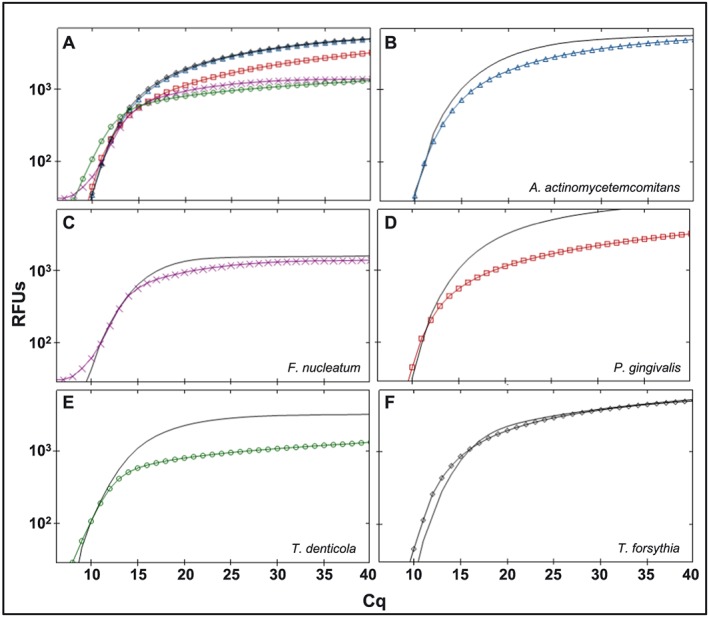
Comparison of multiplex and singleplex assays with TaqMan probe‐based detection. (a) multiplex amplification with mixed TaqMan oligos, mixed TaqMan probes, and mixed target DNA as template. (b–f) Singleplex TaqMan amplification signals when species‐specific TaqMan oligos, species‐specific TaqMan probes, and specific target DNA were used (lines with markers) relative to multiplex amplification signal when mixed TaqMan oligos, mixed TaqMan probes, and mixed target DNA were used (straight lines)

Once the oligo specificities were ascertained, the TaqMan probes were included to optimize a multiplex real‐time amplification condition. In these assays, we included either pure DNA template or a mixture of genomic DNA as template representing all five bacteria of interest. The amplification and *C*_*q*_ values observed with mixed DNA template, mixed TaqMan oligos, and TaqMan probes were similar to the amplification observed with pure single DNA template, corresponding to TaqMan oligo and TaqMan probe (Figure 3 and Table 5). These PCR assays suggested specificity for the TaqMan probes.

**Table 5:** Specificities of TaqMan probes under singleplex and multiplex polymerase chain reaction conditions

Conditions	AA	FN	PG	TD	TF
Singleplex	11.13	12.21	12.20	11.19	12.64
Multiplex	11.28	12.18	12.45	11.26	12.77

*Note*. Numbers indicate *C*_*q*_ values.

AA = *Aggregatibacter actinomycetemcomitans*; FN = *Fusobacterium nucleatum*; PG = *Porphyromonas gingivalis*; TD = *Treponema denticola*; TF = *Tannerella forsythia*.

## 4 DISCUSSION

The current paradigm for evaluating periodontal disease is through several clinical measurements: probing depths, bleeding, attachment loss, vertical/horizontal bone loss, furcation involvement, tooth mobility, and tooth loss (Armitage, 1995). These same clinical measurements are used to determine prognosis and treatment options for patients where the most relied upon clinical parameter is considered the attachment loss (Smiley et al., 2015). However, none of these methods categorize one of the most important risk factors of the disease: the microbiota. Periodontal bacterial composition is not currently being used during the diagnosis of periodontitis, or the determination of the most appropriate therapy. Studies, however, have shown a good correlation between the bacterial markers and progression of the periodontal diseases (Charalampakis et al., 2013; Fernandez y Mostajo et al., 2011; Liljestrand et al., 2014). In addition, there is an agreement of a shift in the microbiota, and individuals with chronic periodontal diseases harbor more anaerobic Gram‐negative bacterial species in their periodontal pockets compared with a population of mostly Gram‐positive facultative anaerobes in the periodontal pockets of healthy individuals (Socransky et al., 1998; Loesche & Grossman, 2001).

In the context of a real‐time multiplex PCR assay, many factors contribute to optimum amplification of target DNA fragments. These factors include the oligo specificities, the quality of the template, amplification conditions, and reagents. In a multiplex real‐time PCR, because multiple targets are amplified with multiple sets of forward and reverse oligos and probes, ideal amplification conditions cannot be met for each target. It is reasonable to adjust PCR conditions in order to attain the most optimum efficiency of amplification for all intended targets while avoiding cross amplification of unintended targets. In the real‐time amplification strategy developed in this study, the amplification efficiency for some bacterial targets is a bit lower. Although this is a limitation of the presented protocol, the oligos demonstrate consistency and reliability in amplification based on the template concentration reflecting the amount of bacterial cells present in any clinical specimen. There was no loss of amplification signal even when DNA specific to five bacterial species targeted in this study was amplified simultaneously compared with when they were amplified individually with pure oligos and genomic DNA.

A variety of techniques have been used for examining the microbial composition in periodontal clinical samples (Eick et al., 2011; Rocas et al., 2015; Zambon & Haraszthy, 1995). These assessments have been invaluable to dental basic science and translational research. Real‐time quantitative PCR (qPCR) has been used as an approach to rapidly quantify specific periodontal pathogens (Masunaga et al., 2010). In addition to real‐time qPCR, other DNA‐based techniques being used in assessing the oral bacteria include checkerboard hybridization (Dahlen et al., 2016; Goncalves et al., 2016; Vieira Colombo et al., 2016). This technique compared with a real‐time qPCR is more labor intensive and time‐consuming. Another limitation of checkerboard hybridization is the detection limit that requires the presence of about 10^4^ to 10^6^ bacterial cells in most samples (Brito et al., 2007). However, with this approach, the presence of a larger microbial population can be analyzed simultaneously (Dahlen et al., 2016; Goncalves et al., 2016; Vieira Colombo et al., 2016). The benefits of a real‐time qPCR, on the other hand, are that it offers a sensitive means of detecting and quantifying small number of bacteria in clinical samples. The drawbacks to this method are the significant cost of qPCR reagents and the time required to set up each assay. Samples often contain small number of bacterial cells, and splitting up the samples to measure separate targets by running independent real‐time PCR assays would be less feasible. Several notable studies in the past have used multiplex PCR as a way to detect more than one pathogen in a sample (Eick & Pfister, 2002; Garcia et al., 1998; Squeri et al., 2006; Tran & Rudney, 1999). However, these traditional multiplex PCR reports failed to provide quantitative data (Eick & Pfister, 2002; Garcia et al., 1998; Squeri et al., 2006). The presence or absence of periodontal pathogenic species without any quantitative data can be misleading because they are often found in low numbers in healthy subjects (Griffen et al., 1998).

The real‐time multiplex PCR offers the advantage of detecting and quantifying specific DNA targets. One of the limitations, however, is the number of bacterial pathogens that can be investigated in a single assay. Considering there are over 600 different bacterial species that make up the oral microbiome (Dewhirst et al., 2010), complete characterization of all these species utilizing the next‐generation DNA and RNA sequencing methodologies might in the future become a more attractive tool. These technologies are currently expensive and far from being accessible for routine analysis of the samples by most clinical laboratories. However, these high‐throughput techniques that assist in rapid characterization of the oral microbiome in health, diseases, and after treatments will likely be a crucial factor in the future in improving overall oral health.

In conclusion, the multiplex real‐time PCR strategy developed in this study can help in assessing relative abundance of five bacterial species that are relevant to oral health. Clinical samples are currently being collected from healthy and periodontal patients that will be used for the validation of this protocol. It is likely that such assays could also be used in assessing the efficacy of any treatment to reduce the burden of bacterial species, which would be valuable for clinical translational research.
